# Chirality inversion in cholesteric phases of bent-shaped liquid crystal dimers with chiral dopants

**DOI:** 10.1039/d5ra06362k

**Published:** 2025-11-19

**Authors:** Yuki Arakawa, Junji Watanabe

**Affiliations:** a Department of Applied Chemistry and Life Science, Graduate School of Engineering, Toyohashi University of Technology 1-1 Hibarigaoka, Tempaku-cho Toyohashi Aichi 441-8580 Japan arakawa@tut.jp; b Laboratory for Future Interdisciplinary Research of Science and Technology, Institute of Science Tokyo Yokohama Kanagawa 226-8501 Japan

## Abstract

Incorporating molecular chirality into liquid crystals (LCs) yields nanoscale one-handed helical structures. The helical pitch of a cholesteric (Ch) phase (*i.e.*, a chiral nematic phase) is typically slightly temperature-dependent, and the sense of helical rotation (*i.e.*, chirality) rarely inverts. This study reports helical pitch divergence and chirality inversion in the Ch phase of bent-shaped LC dimers added with a chiral dopant (CD). We prepared four chiral LC mixtures containing equal proportions of two ester-linked 4-(*trans*-4-pentylcyclohexyl)phenyl-based bent-shaped LC dimers with odd-numbered spacers, each doped with 0.25, 0.50, 1.0, or 2.0 wt% of an isosorbide-based CD, ISO-(6OBA)_2_. All mixtures exhibited enantiotropic chiral twist-bend nematic 
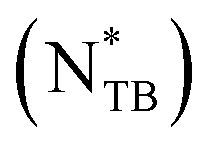
 and Ch phases near room temperature. Polarized optical microscopy revealed that in the Ch phases of the dimer mixtures with 0.25, 0.50, and 1.0 wt% CD, the helix diverged and reformed with opposite chirality just above the 
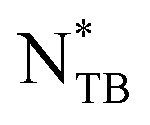
 phase transition temperature. The high- and low-temperature Ch phases were identified as right- and left-handed, respectively, using circular dichroism spectroscopy and the contact preparation method. The dimer mixture with 2.0 wt% CD exhibited only helix divergence of the right-handed Ch phase. These phenomena were also observed in each nonblended dimer, but not in other bent-shaped cyanobiphenyl-based twist-bend nematogenic dimers with different linkages. Our findings offer new insights into chirality inversion in LCs and have promising potential for temperature-tunable chiral-switching systems based on bent-shaped LC dimers.

## Abstract 2

We report helical pitch divergence and chirality inversion in cholesteric phases of bent-shaped liquid crystal dimers added with a chiral dopant.

## Graphical abstract

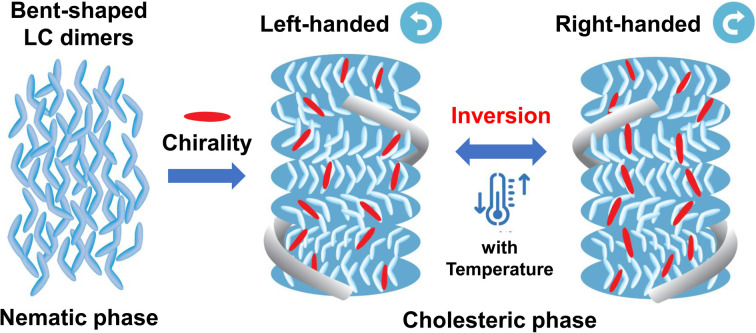

## Introduction

Liquid crystals (LCs), which combine fluidity and anisotropy, enable the facile fabrication of complex helical structures, particularly when coupled with molecular chirality. Not only LC molecules with chiral moieties but also LC mixtures of achiral LC hosts added with a chiral dopant (CD) can spontaneously form chiral LC phases with nanoscale one-handed helices, such as cholesteric (Ch), blue, and chiral smectic C (SmC*) phases. Among such chiral LC phases, the Ch phase (*i.e.*, a chiral nematic phase) is particularly widely explored owing to its versatility and ease of preparation.^1–3^ This phase features one-handed helicoidal structures formed by the rotation of molecular directors with pitches ranging from a few hundred to several thousand nanometers. The helical structure shows Bragg reflection bands for circularly polarized light with the same handedness as the helix, enabling the selective reflection, transmission, and confinement of the incident light. In addition, the fluid Ch alignment and helical pitch can be tailored for specific applications. Therefore, the Ch phase is important for developing various functional and photonic materials in many forms, including films, fibers, gels, droplets, emulsions, and microspheres.^4–18^

Typically, the helical pitches of Ch phases change slightly with temperature, and the sense of helical rotation (*i.e.*, chirality) does not invert.^19^ However, rare examples of temperature-induced helix inversion have been observed in the Ch phases of helical LC polymers^20–23^ and low-molecular-weight calamitic and discotic LCs.^24–36^ This chirality inversion in Ch phases is generally associated with temperature-driven conformational changes in the constituent molecules, which affect the populations of nonequivalent conformers with opposite helical twist senses.^25,27,37–39^ Ch chirality inversion can also be induced by the isomerization of molecules through ultraviolet (UV), visible, and near-infrared light irradiation^14,40–45^ and by changing the concentration of lyotropic LCs and LC mixtures.^37,46,47^ A similar chirality inversion occurs in layered ferroelectric SmC* phases.^48–51^ Chirality inversion in LCs is promising for various photonic applications, beyond LC displays.^6,8,52^

Bent-shaped LC dimers are a distinct class of LCs capable of forming various complex phases, including helical, polar, and frustrated structures.^53–59^ In addition, chirality incorporation into bent-shaped LC dimers can generate twist grain boundaries and stabilize blue phases.^60–66^ The past decade has seen a spurt in research on bent-shaped LC dimers owing to the discovery of a new helical LC phase, namely, the twist-bend nematic (N_TB_) phase.^67–69^ The N_TB_ phase is exclusively formed by achiral bent-shaped molecules and features heliconical structures with degenerated right- and left-handed chiral senses^70^ and short pitches of ∼10 nm.^69,71,72^ Compared to other chiral LC phases, less attention has been paid to chiral N_TB_
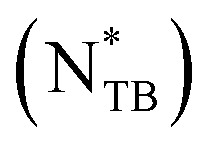
 in terms of synthesizing chiral molecules,^73–79^ adding CDs,^80–82^ and applying molecular theory.^83^ Recently, Ožegović *et al.* reported bent-shaped LC dimers with chiral spacers that exhibit 
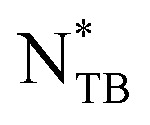
 and Ch phases.^75–77^ Among them, the Ch phases of some naphthyl-containing chiral dimers were found to exhibit temperature-induced chirality inversion,^75^ whose molecular class differs from those of previously reported polymeric and low-molecular-weight Ch materials. Thus, connecting bent-shaped LC dimers to chirality can offer new insights into chirality inversion and switching systems.

**Fig. 1:**
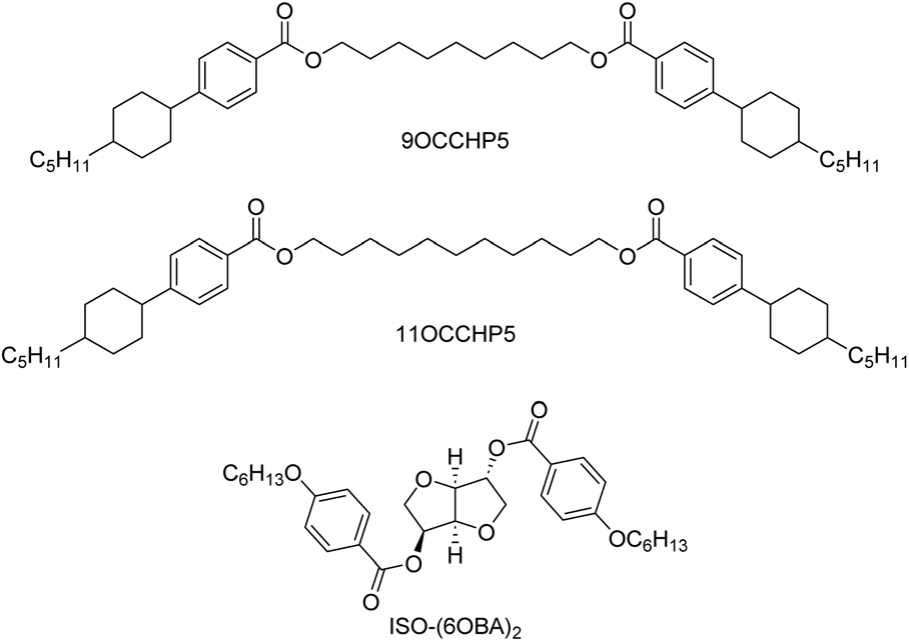
Molecular structures of 9OCCHP5, 11OCCHP5, and ISO-(6OBA)_2_.

Herein, we report temperature-induced helical pitch divergence and chirality inversion in the Ch phases of achiral bent-shaped LC dimers doped with a CD. Unlike the recently reported chiral LC dimers,^75^ the LC dimers employed in the present study are achiral. We examined four chiral LC mixtures containing equal proportions (in wt%) of two ester-linked 4-(*trans*-4-pentylcyclohexyl)phenyl-based LC dimers with nonylene and undecylene central spacers (9OCCHP5 and 11OCCHP5, respectively) and different concentrations (0.25, 0.5, 1.0, or 2.0 wt%) of an isosorbide-based CD, ISO-(6OBA)_2_ (Fig. 1). The 9OCCHP5/11OCCHP5 LC mixture (1 : 1 w/w) exhibits enantiotropic N and N_TB_ phases near room temperature.^84^ The variations in the helical pitch and handedness of the Ch phases in the four mixtures were investigated.

## Experimental

The LC dimers, *viz.*, 9OCCHP5 and 11OCCHP5 were synthesized *via* an S_N_2 reaction between 4-(*trans*-4-pentylcyclohexyl)benzoic acid and 1,9-dibromononane or 1,11-dibromoundecane, as described in the literature.^84^ ISO-(6OBA)_2_ was synthesized from isosorbide and 4-hexyloxybenzoic acid *via* condensation esterification. The cyanobiphenyl-based dimer CB9CB was synthesized according to a similar procedure reported in the literature.^85^ Additionally, the thioether-linked dimer CBS7SCB was synthesized as previously reported.^86^ The molecular structures of the synthesized molecules were determined by NMR spectroscopy using a JEOL JNM-ECS400 spectrometer at 400 MHz (^1^H) and 100 MHz (^13^C). For preparing the LC dimer mixtures, ISO-(6OBA)_2_ (0.25, 0.5, 1.0, or 2.0 wt%) was added to 5 mL glass tubes containing equal weights of 9OCCHP5 and 11OCCHP5. Then, additive-free tetrahydrofuran (THF) was added to dissolve the solids, and the resulting transparent solution was ultrasonicated for 1 min. Thereafter, the solvent was slowly evaporated using a rotary evaporator, and the residue was dried under reduced pressure. Before polarized optical microscopy (POM) observation, the solid mixtures were heated and maintained at 100 °C for at least 10 min to remove residual THF and ensure homogeneity. POM was performed using an Olympus BX53M optical microscope. The sample temperature was controlled with a Mettler Toledo HS82 hot-stage system. Finger print (striation) textures in the Ch phases were well developed in non-treated glass cells with ∼50–100-µm thickness, and helical pitches were determined by measuring the distance between two striations using ImageJ software.^87^ For the contact preparation test, a right-handed Ch standard mixture was prepared by mixing JC-5022X (JNC Co.) with 1 wt% of ISO-(6OBA)_2_. Differential scanning calorimetry (DSC) was performed using a Shimadzu DSC-60 Plus instrument with heating and cooling cycles at a rate of 1 °C min^−1^ under a nitrogen gas flow (50 mL min^−1^). The instrument was calibrated using indium. Circular dichroism spectroscopy for the Ch phase was performed using a JASCO J-1500 spectrometer. The 0.25 wt%-CD-doped dimer sample was placed between two quartz plates, and its homogeneous alignment with the helical axes perpendicular to the cell surface was examined *via* POM. The Ch sample cell was placed in a Peltier cuvette holder with a cell spacer, and the spectra were recorded during cooling from the Iso phase under a nitrogen atmosphere. UV-visible absorption spectra for 9OCCHP5 and 11OCCHP5 were recorded in THF at the ambient temperature using a JASCO V-630 UV-visible spectrophotometer.

## Results and discussion

**Fig. 2:**
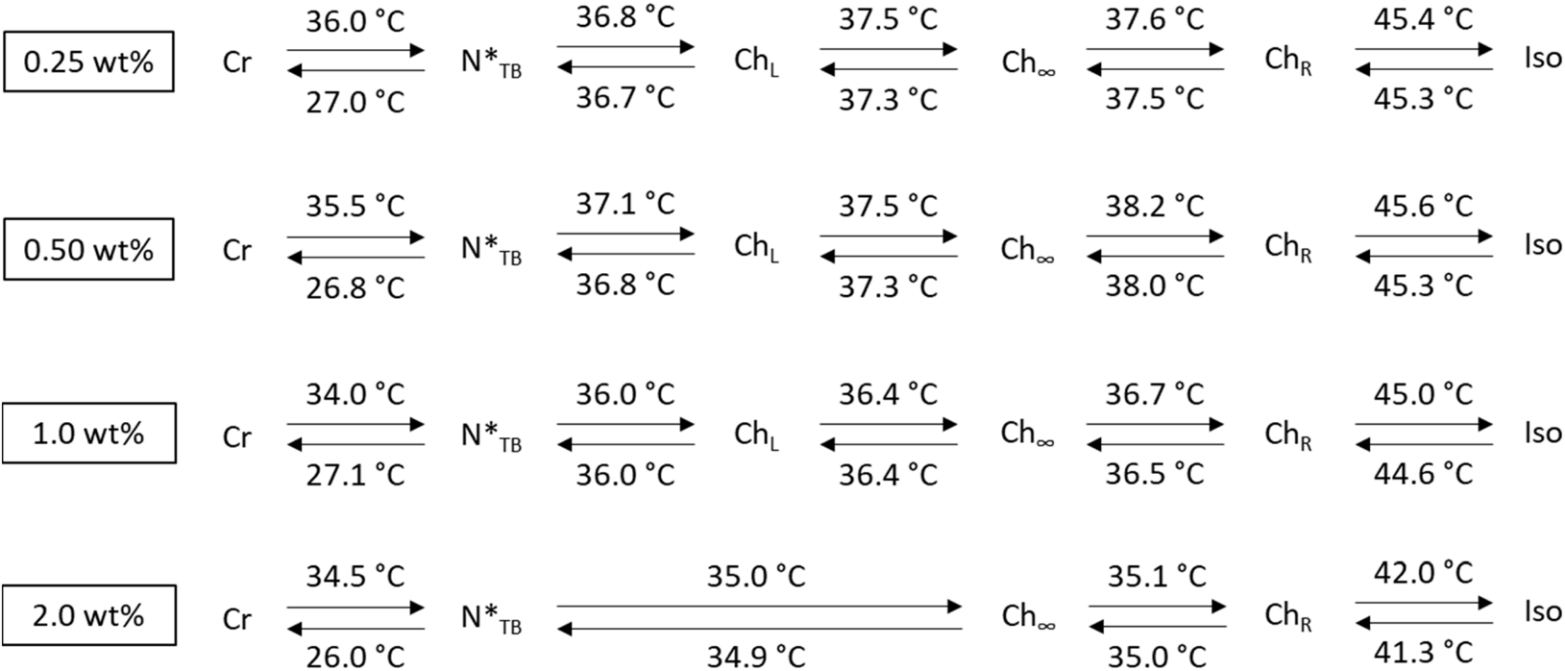
Phase transitions of four 9OCCHP5/11OCCHP5 dimer mixtures with 0.25, 0.50, 1.0, and 2.0 wt% ISO-(6OBA)_2_.

The four 1 : 1 wt/wt 9OCCHP5/11OCCHP5 dimer mixtures with different ISO-(6OBA)_2_ concentrations exhibited the same enantiotropic crystal (Cr)–
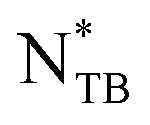
–Ch–isotropic (Iso) phase sequence. This phase sequence is consistent with that of the achiral 9OCCHP5/11OCCHP5 mixture.^84^ However, in the present study, we observed temperature-induced helical pitch divergence and chirality inversion in the Ch phases. The phase transitions of the four mixtures are summarized in Fig. 2, where Ch_R_, Ch_L_, and Ch_∞_ denote the right-handed, left-handed, and pitch-diverged Ch phases, respectively. The Ch_∞_ state corresponds to a conventional nematic (N) phase. The phase transition temperatures were similar for the 0.25 and 0.5 wt% CD-containing LC mixtures, likely because of their particularly low CD content, and they decreased when the CD concentration increased to 1.0 and 2.0 wt%.

**Fig. 3:**
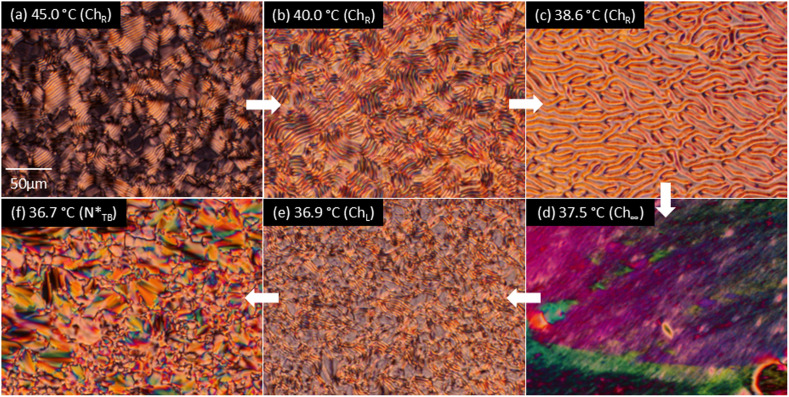
Optical textures of the Ch and 
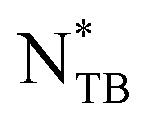
 phases of the 9OCCHP5/11OCCHP5 dimer mixture with 0.25 wt% ISO-(6OBA)_2_ at (a) 45.0, (b) 40.0, (c) 38.6, (d) 37.5, (e) 36.9, and (f) 36.7 °C.

**Fig. 4:**
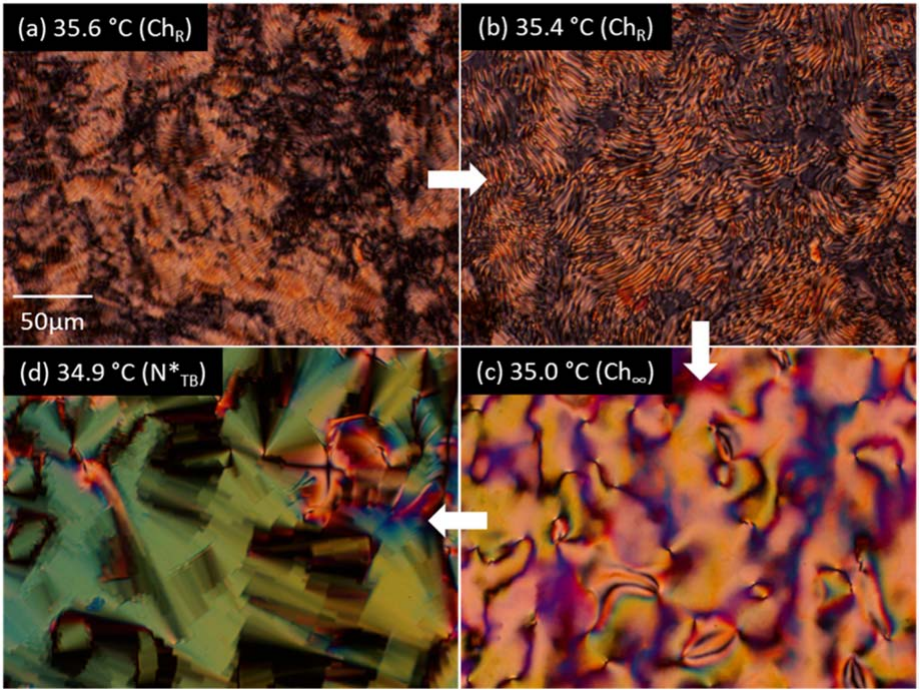
Optical textures in the Ch and 
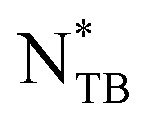
 phases of the 9OCCHP5/11OCCHP5 dimer mixture with 2.0 wt% ISO-(6OBA)_2_ at (a) 35.6, (b) 35.4, (c) 35.0, and (d) 34.9 °C.

Fig. 3 displays the optical textures of the LC phases during the cooling of the dimer mixture with 0.25 wt% CD. The striation (fingerprint) textures indicate that the Ch helix axis lies parallel to the glass surface. Upon cooling, the striation widths increase [Fig. 3(a–c)], and diverge at approximately 37.5 °C, producing a marble texture characteristic of a conventional N phase [Fig. 3(d)]. The Ch striations re-emerge upon further cooling to approximately 37.3 °C [Fig. 3(e)]. This sequence is observed at temperatures within 1 °C above the 
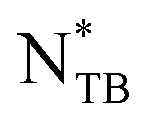
 phase transition temperature [Fig. 3(f)]. Similar texture changes are observed in the 0.50 and 1.0 wt% CD-containing mixtures (Fig. S1 and S2). The 2.0 wt% CD-containing mixture exhibits only helical pitch divergence as shown by Schlieren textures at approximately 35.0 °C, which then transitions to the 
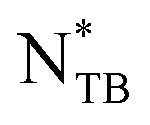
 phase with blocky and focal conic textures (Fig. 4). The DSC curve does not show any changes to indicate the helical pitch divergence and chirality inversion (Fig. S3). Although temperature-dependent pitch variation was also observed in the Ch phase of each nonblended dimer (9OCCHP5 or 11OCCHP5) doped with ISO-(6OBA)_2_, their monotropic LC nature and crystallization during POM observation hindered the analysis of the single-dimer materials. Accordingly, the dimer mixtures were investigated in detail. In addition, we examined bent-shaped cyanobiphenyl-based twist-bend nematogenic dimers with methylene and thioether linkages (CB9CB and CBS7SCB, respectively) doped with 1 wt% ISO-(6OBA)_2_. However, the Ch phases of these materials showed no apparent temperature-dependent pitch variation or chirality inversion, which is consistent with previous reports on chiral LC phases of similar dimers doped with CDs.^80–82^

**Fig. 5:**
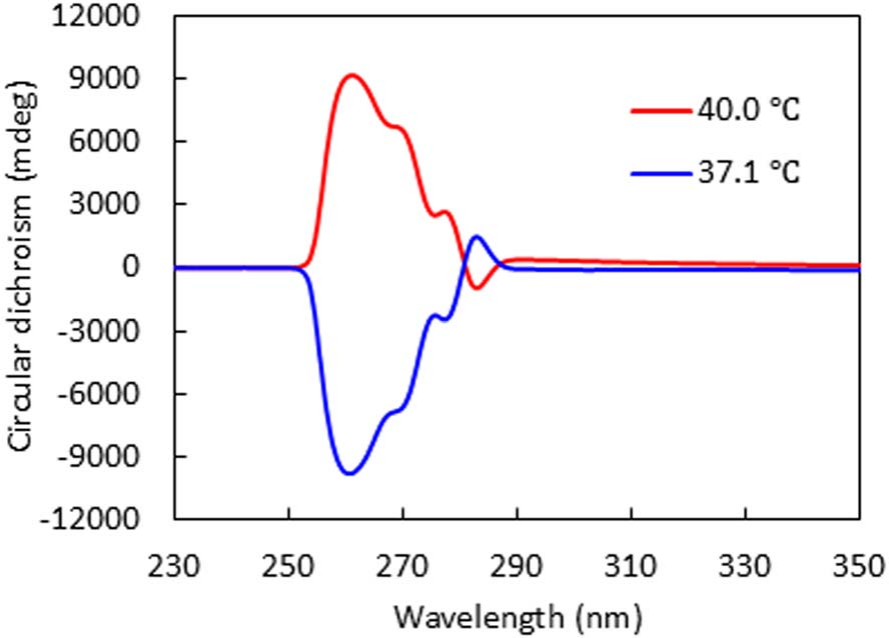
Circular dichroism spectra of the dimer mixture with 0.25 wt% ISO-(6OBA)_2_ at 37.1 °C (blue) and 40.0 °C (red).

To determine the handedness of the Ch phases above and below the chirality inversion temperature, we performed circular dichroism spectroscopy. The 0.25 wt% CD-containing mixture was selected because it had the widest low-temperature Ch-phase window among the mixtures. As shown in Fig. 5, the 0.25 wt% CD-containing mixture shows circular dichroism spectra at ∼250–290 nm at the Ch phase temperature, which was induced for absorption bands of benzoate moieties at ∼240–280 nm (Fig. S4). For the high-temperature Ch phase, the spectrum exhibits a positive Cotton effect, indicating a right-handed helical sense (Ch_R_ phase).^21,59^ Upon cooling into the lower-temperature Ch phase, the spectrum exhibits a negative Cotton effect, indicating a left-handed helical sense (Ch_L_ phase).

**Fig. 6:**
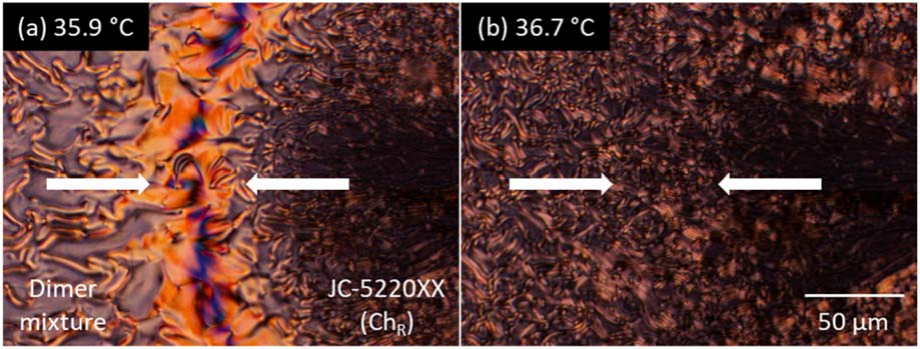
Results of the contact preparation test. Optical textures at the contact boundary between the 9OCCHP5/11OCCHP5-based Ch mixture (left) and right-handed JC-5022XX-based standard (right), both doped with 1 wt% ISO-(6OBA)_2_: (a) at 35.9 and (b) 36.7 °C for low- and high-temperature Ch phases, respectively, of the dimer mixture.

In addition, a contact preparation test was conducted using a right-handed Ch standard of JC-5022XX (formerly JC-1041XX) added with Iso-(6OBA)_2_.^88^Fig. 6 shows the optical textures at the contact boundary between the 9OCCHP5/11OCCHP5 dimer mixture (left) and the right-handed JC-5022XX standard (right), both of which were doped with 1 wt% Iso-(6OBA)_2_. In the low-temperature Ch phase of the dimer mixture [Fig. 6(a)], both textures appear discontinuous at the boundary. This discontinuity arises from a mismatch in the helical sense between the two Ch phases, resulting in an achiral conventional N-like region at the boundary. Therefore, the low-temperature Ch phase has the opposite helical sense to the right-handed standard and is assigned as left-handed (Ch_L_ phase). In the high-temperature Ch phase of the LC dimer mixture [Fig. 6(b)], on the other hand, the textures are continuous across the boundary, indicating identical chiral senses. Consequently, the high-temperature Ch phase of the dimer mixture is assigned as right-handed (Ch_R_ phase). Thus, the chirality signs indicated by the two different methods agree well.

**Fig. 7:**
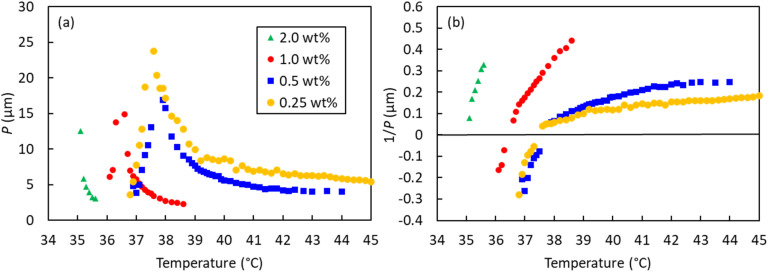
(a) Helical pitch (*P*) and (b) reciprocal pitch (1/*P*) values of the four dimer mixtures as a function of temperature. In panel (b), positive and negative values indicate right- and left-handed helices, respectively.

The striation widths in the optical micrographs were analyzed to determine the helical pitches of the four CD-containing 9OCCHP5/11OCCHP5 dimer mixtures, and the results are plotted as a function of temperature in Fig. 7(a). In the four mixtures, the Ch pitches increase with increasing temperature from just above the 
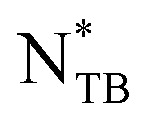
 phase transition temperature, and diverge at the intermediate Ch_∞_ state (N state), except for the 2.0 wt% CD-doped mixture with no Ch_L_ phase. Upon further heating, the pitches of all mixtures decrease and nearly converge upon approaching the Iso phase temperatures. Overall, at comparable temperatures, the helical pitch decreases with increasing CD concentration. This trend is reasonable, because the pitch is inversely proportional to the CD concentration, as follows: *p* = 1/(*β*·*c*), where *p*, *β*, and *c* are the helical pitch (µm), helical twisting power (µm^−1^), and CD concentration (wt%), respectively.

To further examine helix-sense inversion, the reciprocal pitches (1/*p*), which are proportional to the twisting angle between the pseudo-nematic layers in the Ch phase, are plotted against temperature in Fig. 7(b). Positive and negative reciprocal pitch values indicate right- and left-handed helices, respectively. The plots exhibit smooth curves that cross zero, indicating a continuous change in the twisting angle with respect to temperature. The 1/*p* values vary significantly at low temperatures and tend to converge at higher temperatures. The Ch-pitch-diverged temperatures (*T*_Ch∞_) of the mixtures containing 0.25, 0.5, 1.0, and 2.0 wt% CD, determined from the zero crossings of the fitted 1/*p* curves, are 37.5, 37.7, 36.5, and 34.9 °C, respectively. The dimer mixtures with 0.25 and 0.5 wt% CD exhibited similar *T*_Ch∞_ values, but *T*_Ch∞_ decreased as the CD concentration increased from 0.5 to 2.0 wt%, closing to the 
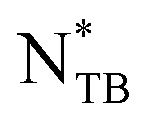
 phase transition temperature. This *T*_Ch∞_ reduction is attributable to the strong twist (shorter pitch) caused by increasing the CD concentration. Ultimately, chirality inversion (formation of the low-temperature Ch_L_ phase) was not observed in the 2.0 wt% CD mixture.

Chirality inversion in the Ch phase is generally linked to a change between the populations of nonequivalent conformers with opposite helical twist senses in the constituent molecules.^25,27,37–39^ In the present study, temperature-induced Ch chirality inversion was observed in the ester-linked dimers, whereas the cyanobiphenyl-based dimers (CB9CB and CBS7SCB) that exhibit the N_TB_ phase showed no such inversion. The ester linkage presents a higher rotational barrier than methylene and thioether linkages; hence, ester-linked dimers are more rigid.^72,89^ This rigidity reduces the possible conformational freedom of the ester-linked dimers, likely leading to nonequivalent conformations that prefer opposite helical twist senses. Ožegović *et al.* reported that bent-shaped LC dimers with a naphthyl ring near the chiral center in the spacer exhibit temperature-induced chirality inversion, whereas phenyl-based analogs do not.^75^ Recently, Ožegović *et al.* also suggested that the naphthyl-based dimer exhibits two stable conformers, which should be responsible for the chiral inversion.^90^ Its naphthyl-based monomeric material also exhibits chirality inversion in the Ch phase. Thus, chirality inversion is not limited to dimeric structures in the case of these naphthyl-based molecules. Therefore, some degree of molecular rigidity, which reduces rotational freedom, can be the key to chirality inversion. In addition, chirality inversion may be linked to some conformational changes in cyclohexylphenyl groups. Zhang *et al.* reported that when added to N hosts as CDs, cyclohexylphenyl-based linear dimers with chiral spacers induced temperature-dependent chirality inversion, whereas their biphenyl variants did not.^32,33^ This result suggests that conformational differences between cyclohexyl and phenyl rings in the CDs may influence the propagation of chirality throughout the LC hosts. For a comprehensive understanding, further study of the chirality inversion of LC dimers is underway in terms of monomer and dimer structures and conformational changes.

## Conclusions

We investigated temperature-induced helical pitch divergence and chirality inversion in the Ch phases of ester-linked cyclohexylphenyl-based bent-shaped LC dimers (*n*OCCHP5) doped with a small amount of Iso(6OBA)_2_ as a CD. These helical changes occurred just above the 
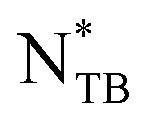
 phase transition temperature. In contrast, typical cyanobiphenyl-based dimers with more flexible linkages exhibited no chirality inversion. Our findings suggest that chirality inversion in ester-linked LC dimers arises from rotationally restricted conformational changes. The chiral inversion of LCs offers immense potential for various optical applications. Accordingly, this study provides valuable insights into the chiral behavior of LCs and easily prepared chiral-switching systems based on LC dimers. Further studies are underway to determine the molecular factors that influence chirality inversion in LC dimers.

## Conflicts of interest

There are no conflicts to declare.

## Supplementary Material

RA-015-D5RA06362K-s001

## Data Availability

The data of this study are presented within the article or supplementary information (SI). Supplementary information is available. See DOI: https://doi.org/10.1039/d5ra06362k.
