# Supplementary material for: Chirality inversion in cholesteric phases of bent-shaped liquid crystal dimers with chiral dopants
Source: RSC Adv. 2025 Nov 19;15(52):44460–6. doi: 10.1039/d5ra06362k (PMC12628302; doi:10.1039/d5ra06362k)
Supplement: RA-015-D5RA06362K-s001 [file RA-015-D5RA06362K-s001.pdf]

Supplementary Information (SI) for RSC Advances.
This journal is © The Royal Society of Chemistry 2025

Supporting information for

Chirality inversion in cholesteric phases of bent-shaped liquid crystal dimers with chiral dopants

Yuki Arakawa *[a] and Junji Watanabe [b]

[a] *Department of Applied Chemistry and Life Science, Graduate School of Engineering, Toyohashi University of Technology, 1-1 Hibarigaoka, Tempaku-cho, Toyohashi, Aichi 441-8580, Japan. E-mail: arakawa@tut.jp*

[b] *Laboratory for Future Interdisciplinary Research of Science and Technology, Institute of Science Tokyo, Yokohama, Kanagawa 226–8501, Japan.*

*Correspondence to Y.A. (arakawa@tut.jp).

POM

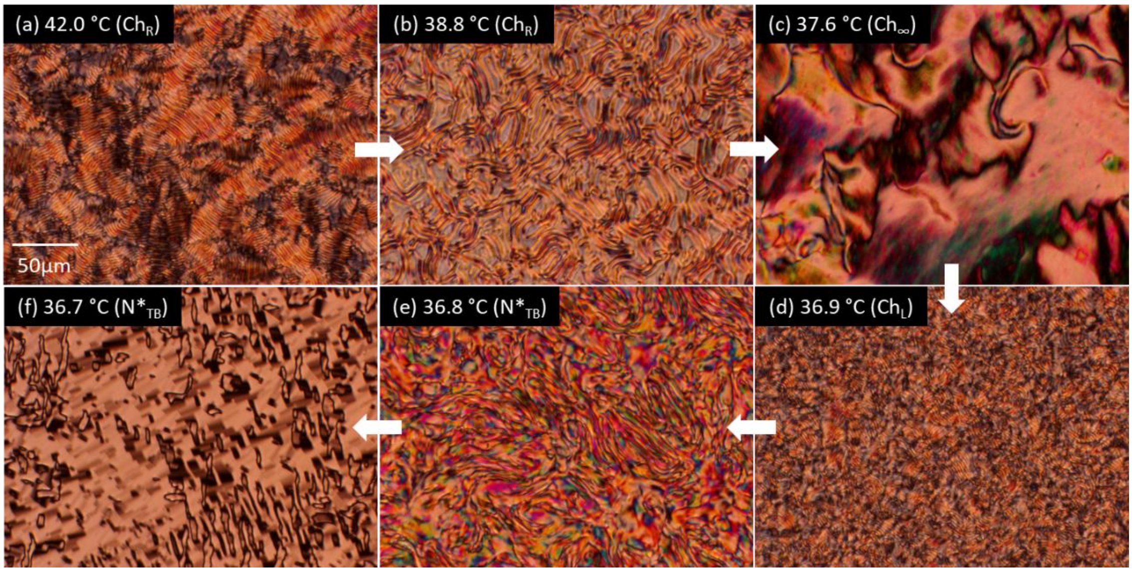

Fig. S1. POM images of the 9OCCHP5/11OCCHP5 dimer mixture with 0.5 wt% ISO-$(6OBA)_2$.

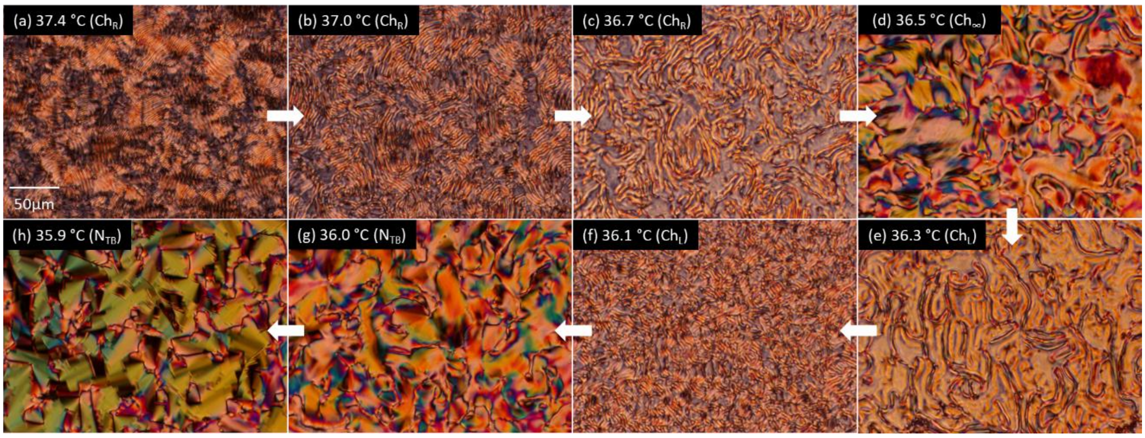

Fig. S2. POM images of the 9OCCHP5/11OCCHP5 dimer mixture with 1.0 wt% ISO-$(6OBA)_2$.

DSC

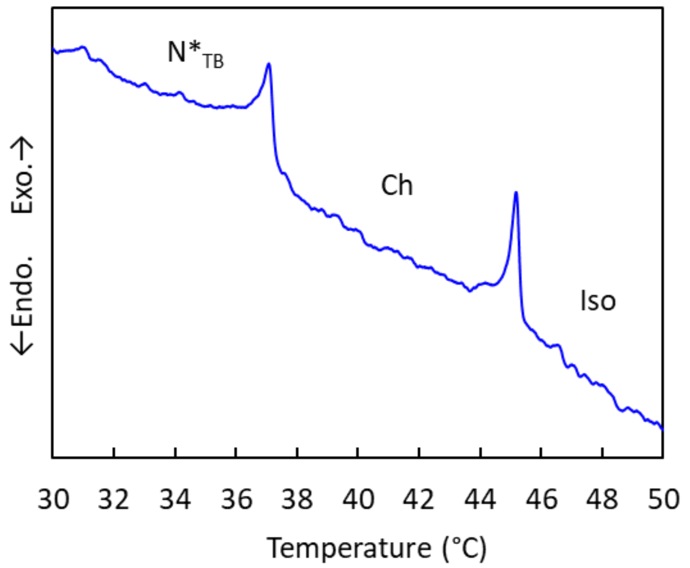

Fig. S3. A DSC curve of the 9OCCHP5/11OCCHP5 dimer mixture with 1.0 wt% ISO-$(6OBA)_2$ at a rate of 1 °C min^{-1}.

UV-Visible absorption spectra

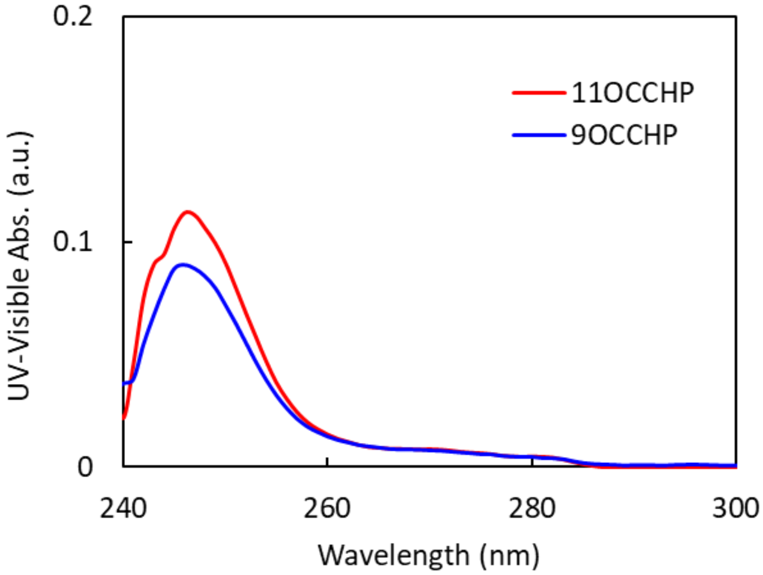

Fig. S4. UV-Visible absorption spectra of 9OCCHP5 (blue) and 11OCCHP5 (red) in THF at the ambient temperature.

Helical pitch plots

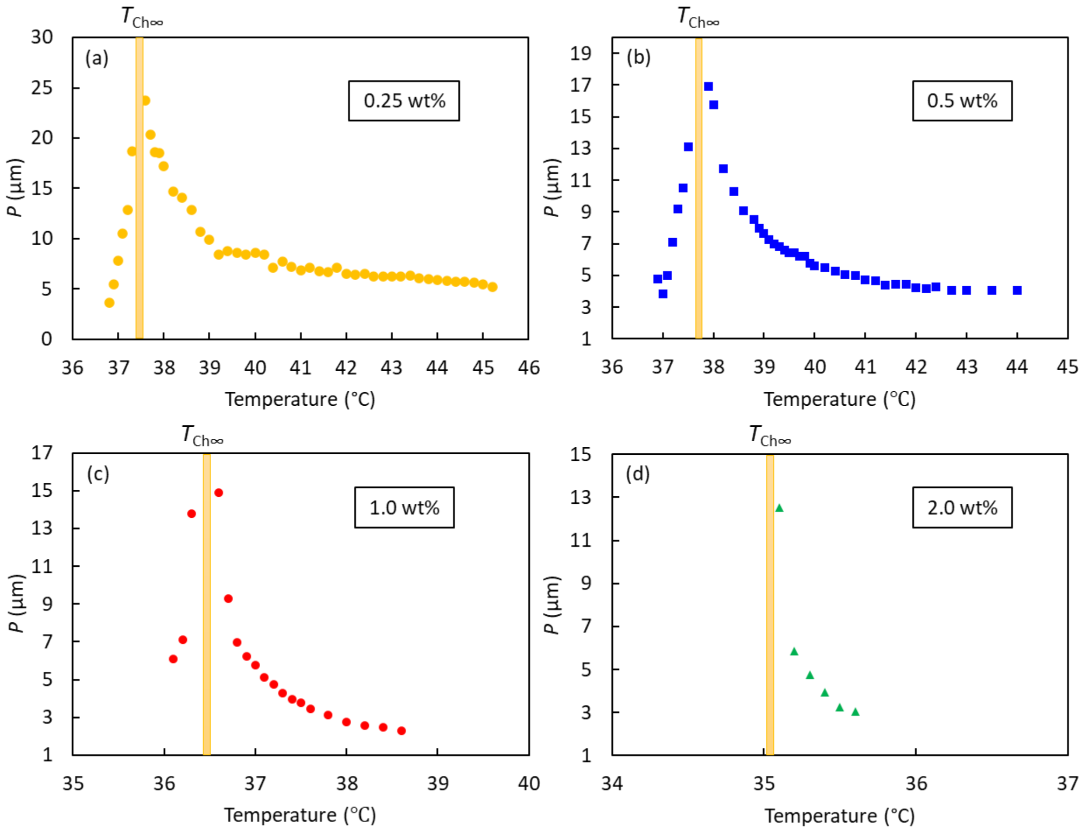

Fig. S5. Helical pitch plots of the four dimer mixtures with (a) 0.25, (b) 0.5, (c) 1.0, and (d) 2.0 wt% CD concentrations.
